# Adenoid Cystic Carcinoma (AdCC): A Clinical Survey of a Large Patient Cohort

**DOI:** 10.3390/cancers15051499

**Published:** 2023-02-27

**Authors:** Mark Zupancic, Anders Näsman, Anders Berglund, Tina Dalianis, Signe Friesland

**Affiliations:** 1 Department of Oncology-Pathology, Karolinska Institutet, 171 64 Stockholm, Sweden; 2 Department of Head-, Neck-, Lung- and Skin Cancer, Theme Cancer, Karolinska University Hospital, 171 64 Stockholm, Sweden; 3 Department of Clinical Pathology, Karolinska University Hospital, 171 76 Stockholm, Sweden; 4 Epistat Epidemiology and Statistics, 752 37 Uppsala, Sweden

**Keywords:** adenoid cystic carcinoma, prognostic factors, subsites, perineural invasion, treatment

## Abstract

**Simple Summary:**

Adenoid cystic carcinoma (AdCC) is a rare heterogenous disease, often difficult to diagnose and prognosticate and, therefore, also challenging to treat optimally. To gain more knowledge with regard to clinical parameters, we conducted a retrospective study on a large cohort of AdCC of the head and neck. The strongest favourable prognostic factors were disclosed to be early disease stage (stage I and II) vs. late disease (stage III and IV) and major salivary gland subsite as compared to other subsites, with the best prognosis in the parotid gland, irrespective of the stage of the disease. Contrary to some studies, we did not find a significant correlation between survival regarding the perineural invasion and radical surgery. However, similar to others, we confirmed that other common prognostic factors, such as smoking, age, and gender, did not correlate to survival and should not be used for prognostication of AdCC of the head and neck.

**Abstract:**

Adenoid cystic carcinoma (AdCC), a rare heterogenous disease, presents diagnostic, prognostic, and therapeutic challenges. To obtain more knowledge, we conducted a retrospective study on a cohort of 155 patients diagnosed in 2000–2022 with AdCC of the head and neck in Stockholm and investigated several clinical parameters in correlation to treatment and prognosis in the 142/155 patients treated with curative intent. The strongest favourable prognostic factors were early disease stage (stage I and II) as compared to late disease (stage III and IV) and major salivary gland subsite as compared to other subsites, with the best prognosis in the parotid gland, irrespective of the stage of the disease. Notably, in contrast to some studies, a significant correlation to survival was not found for perineural invasion or radical surgery. However, similar to others, we confirmed that other common prognostic factors, e.g., smoking, age, and gender, did not correlate to survival and should not be used for prognostication of AdCC of the head and neck. To conclude, in AdCC early disease stage, major salivary gland subsite and multimodal treatment were the strongest favourable prognostic factors, while this was not the case for age, gender and smoking nor perineural invasion and radical surgery.

## 1. Introduction

Adenoid cystic carcinoma (AdCC) is a rare malignancy originating from secretory glands with yet unknown aetiology and accounts for approximately 1% of malignant head and neck tumours [1,2,3]. It is mainly found in the major and minor salivary glands, there accounting for approximately 30% of malignant salivary gland tumours [1,3]. However, AdCC can also arise in secretory tissue in other areas of the head and neck region and, more rarely, also in secretory glands outside this area, e.g., in the oesophagus, breast, lung, prostate, and vulva [1,2,3].

AdCC can occur at all ages but is most common later in life (Fifth to Sixth decade) [1,2]. It frequently presents with unspecific symptoms, slow growth, the perineural invasion, and it is often problematic to diagnose because of the difficulty in defining its clinical differentiation and distinguishing it from benign tumours [1,2,4]. Moreover, there is a lack of good prognostic markers [1,2,4], and conventionally used clinical prognostic markers, e.g., gender, age, or smoking status, are less reliable in AdCC [5]. AdCC is usually treated with surgery upfront, often followed by postoperative radiotherapy, but despite an aggressive therapy regimen late (>5 years after primary treatment), local relapse (15–85%) and distant metastases (25–55%) are common [1]. Due to its slow growth, the disease often runs an indolent course, with 5-year survival rates of 80–85%, but contrary to other head and neck cancers, these promising numbers decline when following 10- and 15-year survival rates, which are 50–60% and 30–35%, respectively [6,7].

Prospective clinical trials are uncommon, but many retrospective studies have shown that disease-free survival (DFS) is better when multimodal therapy is given [2]. One report showed that patients treated with surgery as well as radiotherapy had a much better 5-year local control rate than those given only surgery [8,9]. Another study compared radiotherapy alone with a combination of surgery and radiotherapy; moreover, they also found that local control rates were better with multimodal treatment [10,11]. However, even if DFS is prolonged with combination therapy upon a 5-year follow-up, its effect on long-term (10–15 years) DFS and overall survival (OS) may differ, which in turn is due to the very long follow-up time needed depending on the slow progression of AdCC [11,12]. Moreover, there are no standard recommendations on systemic chemotherapy (ChT) in AdCC patients, and specific ChT regimens have not been proven effective in clinical trials [2]. It has, however, been suggested that the lack of effect of cytotoxic agents is related to the very slow-growing biology of this tumour, but interestingly even in the spread and progressive disease, the treatment response to ChT is very limited (objective response < 20%) [13]. Nonetheless, although not effective, ChT is still being used due to the lack of other effective treatment options for spreading disease [2,13].

Importantly, however, AdCC is a rare and heterogenic disease, and many of the studied cohorts have been small, and moreover, since clinical studies are rare, it has been difficult to draw general conclusions. For this reason, here, we attempted to collect a large cohort of AdCC patients over more than a 20-year period to investigate clinical characteristics and long-time survival in AdCC patients originating from the head and neck region.

## 2. Materials and Methods

### 2.1. Patient’s Characteristics

Patients and tumour samples. Through the Swedish Cancer Registry, between 2000 and 2022, in total, 155 patients were identified as diagnosed with AdCC within the head and neck region (ICD-10: C00.5, C01.9, C04.9, C05.9, C06.9, C07.9, C08.0, C09.9, C11.9, C30.0, and C31.9) in the County of Stockholm/Gotland. Following the identification of these 155 patients in the Swedish Cancer Registry, we could successfully collect and examine the individual charts of all 155 patients at Karolinska University Hospital.

Patients’ charts (collected prospectively during regular contact with the patients during the follow-up period) were assessed. Their tumours were reclassified according to the 8th Edition UICC TNM classification of Malignant Tumours, and age, gender, smoking, performance status (Eastern Cooperative Oncology Group, ECOG), perineural growth pattern, type of treatment, recurrence, time to recurrence and location of recurrence, as well as survival were recorded. Treatment was classified as surgery, radiotherapy (RT) (ablative doses of minimum 64–68 Gy, including both external beam radiotherapy and brachytherapy), chemoradiotherapy (CRT), or palliative therapy (including RT, CRT, and chemotherapy (ChT), but not at curative doses). Smoking data were obtained whenever noted in the charts and were classified as “never” smokers or “ever” smokers (including recent and previous smokers).

All 155 patients were described and characterized according to the criteria above, but 13 were excluded from further survival analyses due to that they did not receive curative treatment or because they were not disease free 6 months after treatment.

Furthermore, for the survival analysis, the 142 patients treated with curative intent were grouped into three subgroups. Most patients (118 cases) were included in the group defined as receiving multimodal treatment and receiving both surgery and RT with or without concomitant chemotherapy (CRT). The remaining patients were divided into two minor groups, one group with 14 cases obtaining only oncological treatment with RT or CRT and one group (10 cases) having surgery alone. DFS was followed through the charts of the patients, while OS was also followed through our access to the Swedish Death Registry from the Karolinska University Hospital.

It is of note that there was no standard follow-up recommendation for AdCC patients and, therefore, in our cohort, all patients did not have the same follow-up period. Nevertheless, the standard follow-up for head and neck cancer (HNC) patients in our institution is clinical controls every 3rd month for the first 2 years and every 6th month until 5 years after treatment. Most patients treated without surgery undergo a computer tomography scan 3 months post-treatment to ensure that there is no residual tumour; the latter is, however, a practice of the more recent years. Most AdCC patients in this cohort continued with clinical controls 2 times yearly after 5 years due to the fact that late recurrences are common. Moreover, the date of death is automatically provided for all citizens that have deceased in Sweden through the digital medical journal system, providing reliable OS data.

The study was conducted according to ethical permissions 99-237, 2005/431-31/4, 2009/1278-31/4, 2012/83-31/2, 2017/1035-31/2, 2019-05211 and 2022-05287-02 from the Ethics Committee at Karolinska Institutet, Stockholm, Sweden, the Stockholm Regional Ethical Review Board, and the Swedish Ethical Review Authority.

### 2.2. Statistical and Survival Analysis

The Kaplan-Meier method was used to estimate overall survival (OS) and disease-free survival (DFS). OS was defined as the time from diagnosis until the date of death of any cause or end of follow-up, whichever comes first. DFS was defined as the time from diagnosis until local or distant recurrence confirmed by radiology or clinical check-up. The chi-square test was used to evaluate differences in categorical data; for continuous variables, an independent two-tailed *t*-test was used. A test result below 5% was considered statistically significant. R version 3.4.1 was used for data management and analysis.

## 3. Results

### 3.1. Patient and Tumour Characteristics

In our cohort of 155 AdCC cases, there was a general predominance of female patients (96 females (F) and 59 males (M); F:M ratio 1.6:1) irrespective of tumour site, with the highest female ratio in AdCC of the parotid gland (F:M ratio 2.5:1) and the lowest female ratio in AdCC of the oral cavity (F:M ratio 1.25:1). For details see Table 1.

In addition, there was a clear predominance of AdCC localized in the major salivary glands (parotid and submandibular gland), the nasal cavity, the paranasal sinus, and the oral cavity (27.1%, 27.1%, 15.5%, and 17.4%, respectively) (Table 1). More specifically, 55.5% of the cases resided in the major salivary glands (if including the two cases of the sublingual gland). Less common tumour sites accounted for 20 cases (12.9%), and these were located in the oropharynx (7 cases, 4.5%), the lip (5 cases, 3.5%), the sublingual gland (2 cases, 1.3%), the larynx (2 cases, 1.3%), the nasopharynx (2 cases, 1.3%), and finally one case each (1 case, 0.65%) was located in the ear and hypopharynx.

A minority of the patients (8/155, 5.2%) presented with spread disease upon diagnosis; furthermore, in one case, the metastasis status was unknown because the patient did not want to undergo further diagnostics due to a high age at diagnosis (90 years old). This patient did not die of AdCC and became 94 years old.

Stage IV disease (including stages IVa, IVb, and IVc) dominated (54 cases, 35.3%), followed by stage II (44 cases, 28.8%), stage I (34 cases, 22.2%), and stage III (21 cases, 13.7%) (Table 1). Male patients tended to present with lower stage; 33/59 (55.9%) stage I and II disease, and 26/59 (44.1%) with stage III and IV, as compared to female patients, where the corresponding proportions were 45/96 (46.9%) and 49/96 (51%), respectively (in two female patients no staging could be assessed). Stage IV was predominant in AdCC in the nasal cavity and paranasal sinuses (17/24, 70.8%) in contrast to the submandibular gland, where only five patients presented with stage IV disease (5/42, 11,9%).

Perineural tumour growth was predominantly found in 100/155 (64.5%) cases, while 52/155 (33.5%) cases presented without a perineural growth pattern and in three cases, the status was not assessable. Perineural growth was over-represented in cases arising from the major salivary glands 62/84 (73.8%), whereas only 38/69 (55.1%) arising from other sites presented with a perineural growth pattern.

In total, 142/155 (91.6%) patients were eligible for treatment with curative intent and were disease-free six months after finishing treatment and included in the further survival analyses. Of the excluded 13 patients, seven had advanced local and/or spread disease at diagnosis, and four were not eligible for intensive treatment due to high biological age and poor performance status. In addition, one patient did not accept treatment, and one showed signs of residual disease after surgical resection and full dose postoperative external beam radiotherapy (not disease-free after a definitive curative treatment regimen).

Out of 142 patients treated with curative intent, 140 presented with locoregional diseases, while 2 had both locoregional and distant diseases.

The latter two patients both had one single metastasis. One patient had metastasis in the liver (primary AdCC in the parotid gland), while the other one had metastasis in the lung (primary AdCC in the nasopharynx). However, both metastases were stable under the curative treatment of the locoregional disease and could later be radically extirpated (negative surgical margins), but unfortunately, both these patients presented with distant recurrence in the lung within three years after finished treatment. A summary of all the patients treated with curative intent is presented in Table 2.

### 3.2. Patients Treated with Curative Intent and Their Clinical Characteristics

The 142 patients treated with curative intent were for the sake of simplicity divided into three subgroups, and the characteristics of these patients are presented in Table 2. The largest group (118 cases) was defined as receiving multimodal treatment and receiving both surgery and RT with or without concomitant chemotherapy (CRT). The second group (14 cases) obtained only oncological treatment with RT or CRT, and the third group (10 cases) had surgery alone.

Of the 118/142 (83.1%) cases treated with multimodal treatment, 89/142 (62.7%) underwent surgery followed by RT, while 29/142 (20.4%) had surgery and postoperative RT (PORT) with concomitant ChT (CRT). Of the 14/142 (9.9%) patients that did not undergo surgery, six received only RT, while eight obtained (CRT). The remaining 10/142 (7%) patients were treated with surgery as a single treatment modality. Below further details per treatment category and patient characteristics are presented.

#### 3.2.1. The Multimodal Treatment Group

This group consisted of 118 patients and was heterogenic, with no subsite or disease stage being overrepresented, suggesting that the multimodal treatment modality was not chosen due to the more advanced disease stage or a certain localization. More specifically, of the 118 patients, 27/118, 22.9% (vs. 34/142, 22.2%) cases were stage I, 38/118, 32.2% (vs. 43/142, 30.3%) stage II, 18/118, 15.2% (vs. 20/142, 14.1%) stage III, and 35/118, 29.7% (vs 45/142, 31.7%) stage IV.

Any recurrence was observed in 39/118 (33.1%) patients. These recurrences were most frequently deriving from primary AdCCs in the nasal and paranasal sinuses 11/18 (61.1%), followed by those in the oral cavity 8/17 (47.1%), the submandibular gland 12/35 (34.3%), 4/14 (28.6%) in other sites, and finally, they were least commonly found 4/34 (11.8%) in AdCC primaries of the parotid gland.

Locoregional recurrence (LRR), occurring in 15 cases (15/118, 12.7%), was most common in the nasal cavity and paranasal sinuses with 8/15 (53.3%) cases, where all but one had initially presented with stage IV disease at diagnosis. Interestingly 11/15 (73.3%) cases with LRR developed even distant metastases. However, only six of these patients died within five years of the diagnosis of LRR.

Distant metastases were developed by 36/118 (30.5%) patients, of which 25/36 (69.4%) were diagnosed only with distant metastases and 11/36 (30.6%) with both LRR and distant diseases (see above). Distant metastasis without LRR was most common among primary AdCC located in the oral cavity 5/17 (29.4%), followed by those located in the submandibular gland 10/35 (28.6%), while the distant disease was less common in primary AdCC at other sites.

The most frequent site of distant metastases was the lungs (25/36, 69.5%), followed by the liver (4/36, 11.1%), which was also the most common site of secondary metastases after the lungs. Other sites of the distant disease include: the bones, the kidneys and the adrenal glands, the brain, the skin, the lymph nodes, and the thyroid gland.

#### 3.2.2. The RT and CRT Group

In this group, the majority 12/14 (85.7%) presented with advanced disease (ten cases with stage IV and two with stage III disease), and 12/14 (85.7%) presented with any late recurrence, of which five cases had LRR, four had both LRR and distant metastasis, whereas two developed distant disease alone.

#### 3.2.3. The Surgery Alone Group

In this group, all 10 cases had the early-stage disease (stage I and II), and the surgery was radical in 8 cases, while in the 2 cases where the surgery was not radical, both patients were recommended PORT, but this was not conducted initially.

One patient undergoing non-radical surgery later presented with locoregional recurrence and was treated with RT, while the other never encountered a recurrence.

Of the eight cases having a radical extirpation of the tumour, one patient still presented with a locoregional recurrence (nasal cavity).

### 3.3. Survival Analysis

Several survival analyses were performed on the 142 patients treated with curative intent, and some of the most notable parameters are presented below.

#### 3.3.1. Long-Term Disease-Free Survival and Overall Survival for Patients Treated with Curative Intent

**Figure 1:**
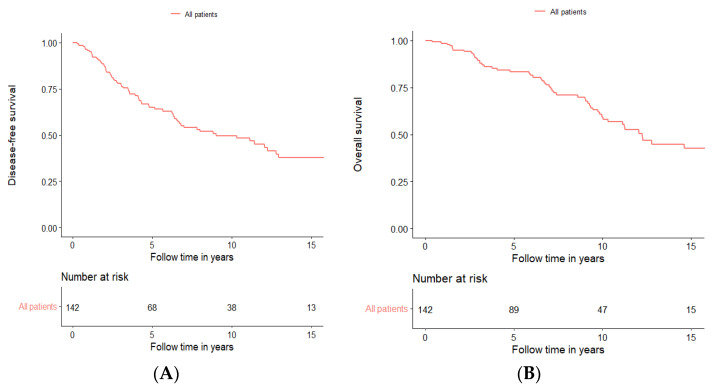
Disease-free survival (**A**) and overall survival (**B**) of all patients treated with curative intent independent of treatment modality.

Initial survival analysis for DFS and OS was performed including all 142 patients treated with curative intent, irrespective of treatment modality. For this cohort, the 5-year DFS was 64.9%, the 10-year DFS was 49.6%, and the 15-year DFS was 37.7%, while the 5-year OS was 83.5%, the 10-year OS was 59.4%, and the 15-year OS was 42.5% (Figure 1A,B, respectively, and Supplementary Table S1).

#### 3.3.2. Long-Term Disease-Free Survival Separated for Different Treatment Modalities

**Figure 2:**
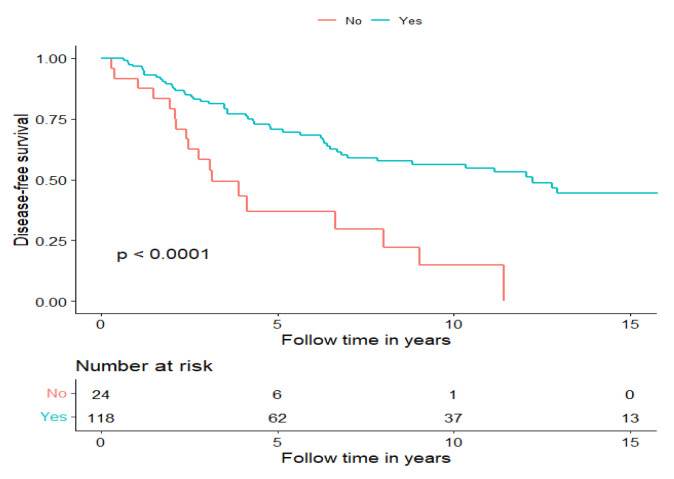
Disease-free survival of patients treated with curative intent separated for treatment modality, multimodal treatment (Yes) and surgery or RT w/o ChT (No).

Patients receiving multimodal treatment had a significantly better DFS compared to patients receiving single treatments, i.e., the 14 patients in the RT and CRT group and the 10 patients receiving surgery alone (log-rank: *p* < 0.0001). More specifically, the five-year DFS was 70.7% in patients treated with multimodal treatment compared to 37% in patients receiving single modality treatment (Figure 2), and similar data were found for OS (Supplementary Figure S1).

#### 3.3.3. Long-Term Disease-Free Survival Separated for Treatment with Surgery and RT, and Surgery and CRT

**Figure 3:**
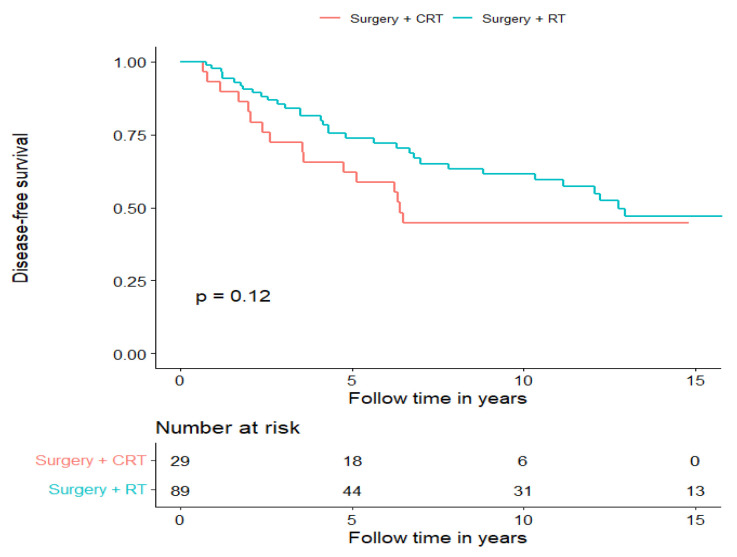
Disease-free survival of patients treated with curative intent separated for treatment modality, surgery + PORT and surgery + postoperative CRT.

To examine whether postoperative CRT improved survival compared to PORT, we compared these groups and found a non-significant tendency of a longer DFS and OS in the PORT group (for DFS, see Figure 3 and for OS, see Supplementary Figure S2). Notably, both groups were heterogenic; however, although there was no clear disproportion of different AdCC subsites, the stages of the disease proportions were imbalanced. Advanced disease was clearly overrepresented in the group receiving postoperative CRT (Stage IV: 13/29, 44.8%, Stage III: 6/29, 20.7%), whereas the group receiving PORT had more early-stage disease cases (Stage I: 24/89, 27%; Stage II: 31/89, 34.8%). Furthermore, in this cohort, we investigated how radical surgery (negative surgical margin in the pathology report) correlated with DFS (recurrence of any kind) and OS; however, there was no significant difference between the groups (data not shown).

#### 3.3.4. Long-Term Disease-Free Survival in Relation to Gender, Age, and Smoking in AdCC

To investigate whether gender, age, and smoking today used as classic prognostic factors in the clinic are prognostic in AdCC, we performed a separate survival analysis for these parameters. For age, we used the median age in our cohort, which was 58.5 years, and we separated the survival analysis for age >58.5 and <58.5 years. For smoking, a dichotomized smoking status (Ever vs. Never) was used.

**Figure 4:**
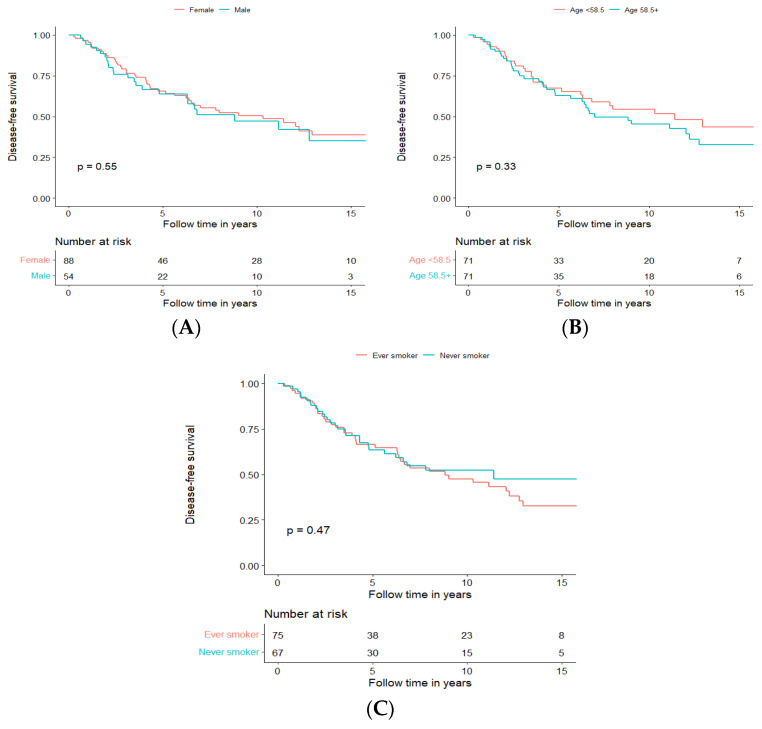
Disease-free survival of patients treated with curative intent independent of treatment modality, depending on gender (**A**), age (**B**), and smoking (**C**).

We could not confirm any of these factors to be prognostic for DFS in our cohort of 142 patients receiving treatment with curative intent, and the data are presented for DFS in Figure 4A–C, respectively, and for OS in Supplementary Figure S3.

#### 3.3.5. Long-Term Disease-Free and Overall Survival for Patients Treated with Curative Intent, Independent of Treatment Modality, in Relation to Perineural Growth Status

**Figure 5:**
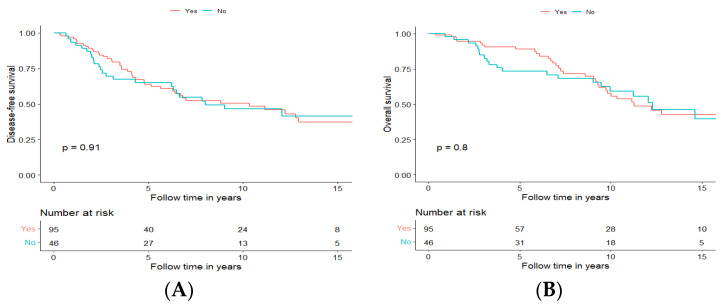
Disease-free survival (**A**) and overall survival (**B**) of patients with tumours with perineural growth (Yes, Orange) and without perineural growth (No, Blue).

Perineural tumour growth was observed in 95 (66.9%) of all cases undergoing curative treatment and was, in general, more frequent in the major salivary glands and most frequent in tumours from the submandibular gland (31/38; 81.6%), followed by the parotid gland 28/41 (68.3%), the oral cavity 16/25 (64%), the nasal cavity and paranasal sinuses 13/21 (61.9%). Perineural invasion was, however, much less common (7/17, 41.2%) in AdCC arising from other sites. Markedly, perineural growth did not always correlate with a higher disease stage since 55.9% of the cases had perineural invasion in stage I, 74.4% in stage II, 80% in stage III and 62.2% in stage IV. Furthermore, it is of note that there were no significant differences in DFS nor OS when separating the analysis for perineural invasion, dichotomizing positive and negative status, according to the pathological report (Figure 5A,B, respectively).

#### 3.3.6. Long-Term Disease-Free and Overall Survival for Patients Treated with Curative Intent, Independent of Treatment Modality, in Relation to Tumour Stage at Diagnosis

**Figure 6:**
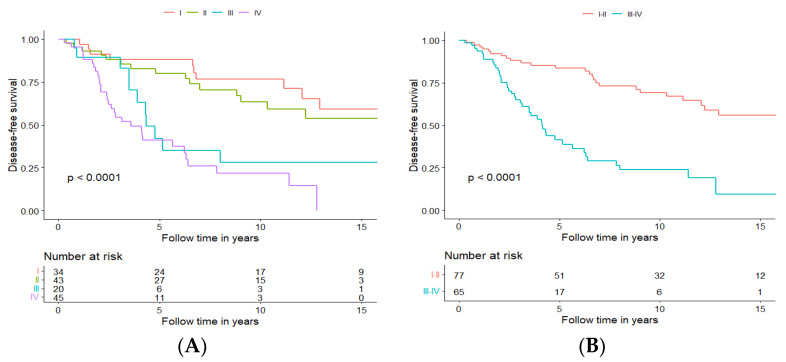
Disease-free survival of patients with tumours staged I–IV (**A**) or with tumours staged I and II vs. III and IV (**B**).

As mentioned above, of the 142 patients, 34 (23.9%) presented tumours in stage I, 43 (30.3%) in stage II, 20 (14.1%) in stage III, and 45 (31.7%) in stage IV. Patients presented with the lower disease stage, treated with curative intent, had a significantly better DFS and OS, with DFS presented in Figure 6A and OS in Supplementary Figure S4A.

After performing the survival analysis for each individual stage, it became clear that stages could be clustered into two groups, with early-stage group clustering stages I and II and advanced-stage group clustering stages III and IV. Stages I and II clustered together had significantly better DFS and OS (DFS presented in Figure 6B, and OS presented in Supplementary Figure S4B); more specifically, five-year DFS and OS were 83.6% and 90.0%, respectively, for the early-stage group, and 41.3% and 75.0%, respectively, for the advanced stage group. In addition, 10-year DFS and OS were 69.2% and 72.3%, respectively, for the early-stage group and 23.9% and 42.7%, respectively, for the advanced-stage group.

#### 3.3.7. Long-Term Disease-Free and Overall Survival in Patients Treated with Curative Intent, Independent of Treatment Modality, in Relation to Most Common Tumour Subsites

To investigate whether primary AdCC within different subsites of the head and neck region have a different prognosis or not, we analysed DFS and OS according to the four most frequent subsites, independent of treatment modality. Altogether 125 cases of primary AdCC, all originating from either the parotid gland (41/125, 32.8%), the submandibular gland (38/125, 30.4%), the nasal cavity, the paranasal sinuses (25/125, 20%), and the oral cavity (20/125, 16%), were included in the analyses.

**Figure 7:**
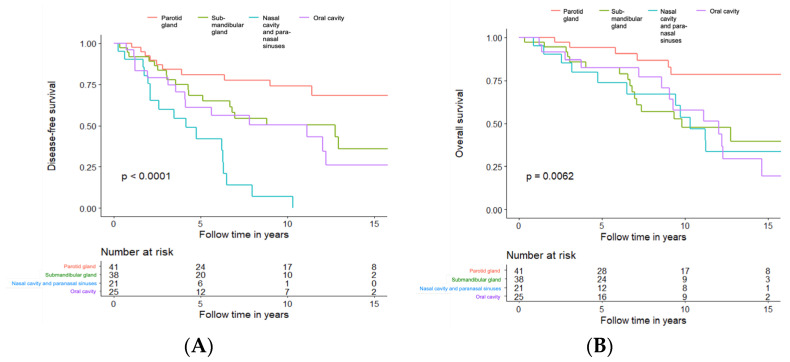
Disease-free survival (**A**) and overall survival (**B**) in patients treated with curative intent, independent of treatment modality, in relation to most common tumour subsites. Tumour subsites: **---** Parotid Gland; **---** Submandibular Gland; **--- ** Nasal Cavity and Paranasal Sinuses; **---** Oral Cavity.

Primaries from the nasal cavity and paranasal sinuses (Subsite C) had the least favourable DFS with a 5-year DFS of 42%, followed by the oral cavity at 61.2% (Subsite D), the submandibular grand 68.3% (Subsite B), and the parotid gland 81.1% (Subsite A), and for details of 10- and 15-year DFS, see Figure 7A.

For OS analysis, the differences between the groups were still significant (log-rank *p* = 0.0062) but less obvious (Figure 7B). The early recurrences of the primaries from the nasal cavity and paranasal sinuses seemed to influence the OS only marginally. The five-year OS for the submandibular gland, the oral cavity and the nasal cavity and paranasal sinuses was very similar (82.4%, 82.5%, and 73.7%, respectively). Primaries from the parotid gland had the best OS (5-year OS of 94.3%), and for details of 10- and 15-year OS, see Figure 7B.

**Figure 8:**
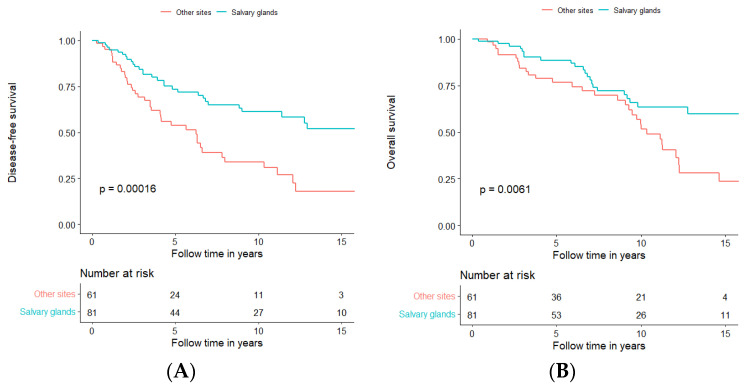
Disease-free survival (**A**) and overall survival (**B**) in patients treated with curative intent, independent of treatment modality, in relation to all tumour subsites, separated for major salivary glands in blue and all other sites together in orange.

Since the prognosis of AdCC in the major salivary glands seemed to be more favourable when compared to that in other sites, a further analysis was performed comparing DFS and OS for all major salivary glands (parotid, submandibular, and sublingual gland) to DFS and OS of all other sites (Figure 8A,B, respectively).

AdCC in major salivary glands had better DFS and OS compared to other sites (log-rank test, DFS: *p* = 0.00016; OS: *p* = 0.0061); more explicitly, five-year DFS and OS were 73.5% and 88.7%, respectively in AdCC of the major salivary glands as compared to 53.7% and 76.8%, respectively of AdCC in other sites. Moreover, 10-year DFS and OS for salivary glands were 61.3% and 63.6%, respectively, compared to 33.8% and 54.2%, respectively, for other sites. For further details and 15-year DFS and OS, see Figure 8A,B and Supplementary Table S1.

#### 3.3.8. Multivariable Analysis for Different Prognostic Factors Used in the Survival Analyses for the Entire Cohort, Treated with Curative Intent

**Figure 9:**
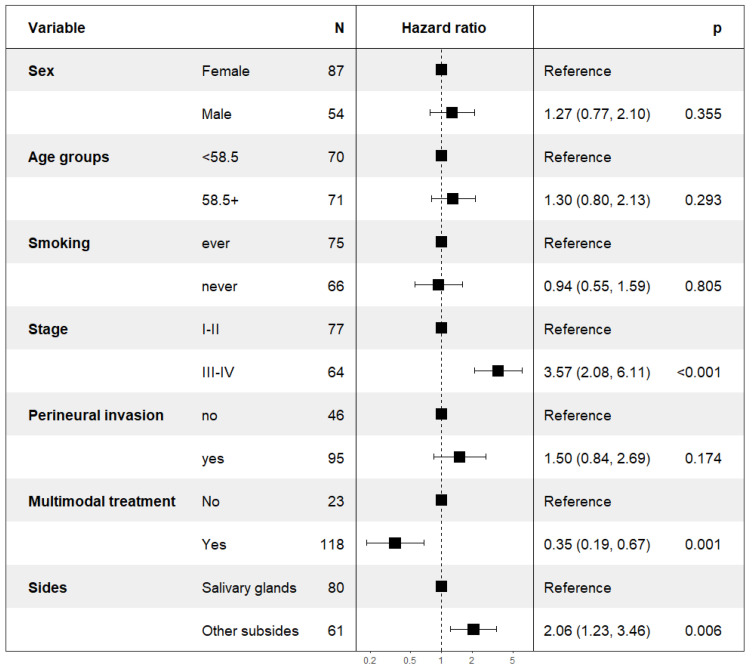
Multivariable analysis of DFS in patients treated with curative intent.

A multivariable subgroup analysis, including all patients treated with curative intent, irrespective of treatment modality, was performed. Multivariable analyses for DFS and OS were performed for gender, age, smoking status, stage of disease (I + II vs. III + IV), perineural invasion, and dichotomizing surgery and RT/CRT vs. surgery or RT/CRT alone, as well as subsites (Salivary glands vs. Other subsides). For DFS, see Figure 9 below and for OS, see Supplementary Figure S5. (Univariable analysis was very similar and is shown in Supplementary Table S2.)

The multivariable analysis confirmed that typical clinical prognostic factors, such as gender, age, smoking status, and perineural growth pattern, were not applicable in AdCC. However, the multivariable analysis supported that the stage of the disease, as well as the subside of the AdCC, could be used as a prognostic factor. In addition, the multivariable analysis supported that combinational treatment of surgery and RT w/o ChT was superior to single treatment modality and resulted in improved survival; for details of DFS, see Figure 9 above and for OS, see Supplementary Figure S5.

## 4. Discussion

In this retrospective study with a follow-up for >20 years, a large cohort of head and neck AdCC patients was examined with regard to clinical characteristics and long-time survival in correlation to gender, age, smoking, perineural invasion, tumour stage, subsite, and treatment modality. We could disclose that both DFS and OS were superior following multimodal treatment modality vs. single treatment modalities. Moreover, patients with tumour stages I and II had a better prognosis than those with stage III and IV disease and those with major salivary gland AdCC had a better outcome than those with AdCC at other subsites, irrespective of disease stage. In contrast, gender, age, smoking status, and perineural growth pattern in the tumour, factors otherwise used for prognostication in many solid cancers [14,15], did not affect either DFS or OS.

Based on the findings above, we first compared our cohort to those of others and found no major differences in the characteristics of patients included in the study as compared to those included in other reports [2,6,16,17]. More specifically, the overrepresentation of female patients (F:M ratio 1.6:1) and the median age of 58.5 years at diagnosis were not unexpected since AdCC patients often are younger than other head and neck cancer patients and AdCC is more common in females [2,9,16,17]. The latter has been discussed to be linked to biological differences between the sexes and different hormonal levels [18], but this probably plays a less important role in the AdCC of the head and neck region.

Moreover, only around 5% of the patients presented with metastatic disease at diagnosis, suggesting, in line with the present literature, that AdCC is mainly a locoregional disease [6,17,19]. Furthermore, similar to other reports, most patients were treated with curative intent, and the majority were treated multimodally with surgery and PORT [2,3,9,16,20].

We then investigated DFS and OS taking into account the whole cohort and independent of treatment modality; similar to others, we also showed that neither gender, age, or smoking influenced DFS, although some studies have shown that older age is associated with higher disease stage and consequently poorer DFS [9,16,17,18]. OS was, however, worse in older patients, both in our cohort as well as in others, which was not really unexpected since these patients were older [7,21].

Notably, however, we found that AdCC in the major salivary gland subsite and lower disease stage were prognostic factors, and these data are presented in more detail below upon also discussing treatment modality.

Combinational treatment (e.g., surgery and PORT) has been suggested to be important for achieving good control of AdCC in general [11,12,22], and both locoregional and distant relapses were shown to be less common in patients treated with a combination of surgery and PORT [9]. In our cohort, this was not different, and we also reported that multimodal treatment correlated significantly with fewer recurrences of any kind. In line with the current literature, during the present observation period, 53/142 (37.3%) patients presented recurrent disease of any kind after curative treatment [23]. Recurrences were most common in the group receiving only RT or CRT (85.7%) and least common in the group treated with surgery and PORT (33.0%).

A large American cohort study compared surgery alone to surgery and PORT and found similar to us that the latter is superior and significantly improves survival [9]. They, however, suggested that the role of PORT needs to be studied further in low-risk patients. Here we showed that surgery alone can be considered in low-stage disease (Stage I–II) when PORT is contraindicated for any reason. Nevertheless, in our cohort, surgery as a single treatment modality was only used in patients with limited disease, but it did accomplish very good local control (one LRR) with no distant disease (10 patients).

Induction chemotherapy (ICT) is, to our knowledge, not commonly used in the treatment of AdCC of the head and neck region and was not often reported in the studies we have cited above [17]. In line with the literature, only five patients in this cohort received ICT; in four cases, a combination of Cisplatin and Fluorouracil and, in one case, even Docetaxel was added. In these cases, the primaries originated from different subsites (parotid-, submandibular gland, oropharynx, oral-, and nasal cavity); four had a stage IV disease, and one case was an early-stage disease. Three patients were even operated on for the primary tumour, and they all received CRT after ICT. ICT did not seem to improve survival in our patients since 3/5 patients presented recurrent disease within two years, while one died of secondary cancer; however, one patient with an early-stage AdCC in the parotid gland is still alive after seven years.

Interestingly, we could not show that radical surgery (negative surgical margins) could be used as a possible prognostic marker, as suggested in other studies [20,24,25]. In fact, in our cohort, we could not show any significant difference in either DFS or OS with regard to if patients underwent radical surgery or not. The latter suggested that PORT successfully treats the possible residual disease after surgery and confirmed its importance for good and long disease control.

The fact that patients with tumour stages I and II had longer survival than those with tumour stages III and IV was not unexpected since this is usually the case for most solid tumours, including AdCC [20,26,27]. The most important part of staging is the size of the tumour (T-stage) [16,28], as AdCC rarely primarily spreads to the lymph nodes [29]. We confirmed that smaller tumours had a better prognosis and that staging should be used as a clinical prognostic factor in AdCC of the head and neck region similar to that for many other tumours as well as AdCC [16,30].

We also showed that subsites could be used as a prognostic factor since major salivary gland AdCC had a significantly better prognosis than AdCC in other subsites, and notably, especially AdCC within the parotid gland had the best prognosis. This has previously been shown in some cohorts but not in all studies since, in other studies, subsite did not influence the prognosis [16,20,25,31,32]. In this AdCC cohort, there was a large proportion of primaries within the major salivary glands (57% of patients treated with curative intent), which is not always the case in the literature [2,9,33]. Moreover, they also had a better prognosis, which is in line with some reports, but different from some other studies [16,20,25,31,32]. Additionally, we showed that AdCC arising in the nasal cavity and paranasal sinuses had the least favourable DFS; however, this did not, to the same extent, influence the OS. These patients were more often diagnosed with advanced disease and had the highest rate of locoregional relapses, which can successfully be treated with, e.g., re-irradiation, achieving good control of the disease. Similar data have also been shown in other cohorts [34,35].

Of note, the frequency of perineural growth in our cohort was in line with that shown previously by others [9,17,33,36]. Moreover, perineural growth was most common in the major salivary glands (especially the submandibular gland), where AdCC had a more favourable outcome compared to other sites in the head and neck. However, the perineural invasion was not linked to a higher disease stage, as one could have expected. Others have shown that perineural growth is associated with positive surgical margins and linked to a higher frequency of LRR or poorer prognosis in general [16,22,33,37,38,39]. Some have also shown that perineural invasion, especially of larger nerves, is linked to distant metastases and, thereby, to a less favourable prognosis [7,16,22]. In contrast to previous findings, none of the latter was the case in our cohort; on the contrary, we found that both LRR and metastatic disease were overrepresented in the group of patients presenting without perineural invasion. Our findings are, therefore, in contrast with a meta-analysis conducted by Ju et al. [37] that indicated that perineural invasion was strongly associated with poor DFS and OS [33,37]. The reason for this discrepancy we do not presently know, but also others have shown similar findings [25]. On the other hand, AdCC is a rare disease with many parameters that have not yet been resolved, so despite the fact that both cohorts are fairly large, the differences could be due to chance or other unknown factors.

Notably, there are some limitations in this study. Firstly, although we have many patients, the study is still a retrospective one, and not all patients were followed for >15 years. Furthermore, patient treatment varied to some extent, although most patients received multimodal treatments. Finally, the follow-ups of the patients were not identical, which potentially could, in some cases, depending on the follow-up, affect the DFS data; still, this is also the case for many other cohorts reported by others. However, this was not the case for the OS data, which were derived through the Swedish death registry.

Other limitations are that we do not include studies of immunohistological or molecular biomarkers in this study, of which some have been described before to be of specific value and where some could be of use for targeted therapies [40,41,42,43,44,45,46]. More specifically, e.g., recurrent rearrangements in *MYB* or *MYBL1* genes, overexpression of *MYB-NFIB* transcripts, or mutations that activate the NOTCH pathway can be of value for prognostication and/or specific therapies [40,41,42,43]. Moreover, the identification of different immunological markers could also be of value [47]. These are all important issues that require specific thorough investigations that we need to follow up on. We have, however, previously investigated the potential role of both human papillomaviruses (HPV) and polyomaviruses in AdCC and could not show that they were associated with the development of AdCC [48]. Moreover, finding HPV in tumours resembling AdCC implied that the diagnosis was not AdCC [48].

In summary, we confirmed that AdCC is more common in female patients; it appears in adult patients, most often in their fifth decade, but can even appear in younger and elderly aged patients. The strongest prognostic factors are the stage of the disease, as well as the subsite, with the best prognosis of AdCC in the major salivary glands. Furthermore, multimodal treatment was superior to single-treatment modalities.

Contrary to what others have shown, we could not find any significant correlation in survival in regard to perineural invasion and radical surgery. Furthermore, we found that other common prognostic factors, such as smoking status, age, and gender, often applicable for other cancer types, should not be used as prognostic factors in the AdCC of the head and neck. This study underlined the need to in the future find new molecular prognostic markers and additional treatment options for this disease and identify patients with poor prognosis upfront since, so far, systemic oncological treatments do not show sufficiently promising results [2,39,48].

## 5. Conclusions

In this retrospective study, we confirmed that AdCC was more common in female patients, often occurring in the fifth decade, although it can appear in younger and older ages. In our cohort, the strongest favourable prognostic factors were early disease stage (stage I and II) and major salivary gland subsite, with the best prognosis in the AdCC of the parotid gland. Furthermore, multimodal treatment was superior to single-treatment modalities.

In contrast to some other reports, we did not report any significant correlation between perineural invasion, radical surgery, and survival.

However, in addition, similar to other studies, we confirmed that other common prognostic factors, such as smoking status, age, and gender, should not be used as prognostic factors in the AdCC of the head and neck region.

## Figures and Tables

**Table 1:** Patients and tumour characteristics, entire cohort.

		Total (%)	Parotid Gland (%)	Submandibular Gland (%)	Nasal Cavity & Paranasal Sinuses (%)	Oral Cavity (%)	Other Sites (%)
Number of patients		155	42	42	24	27	20
Gender	Female	96 (61.9)	30 (71.4)	26 (61.9)	14 (58.3)	15 (55.6)	11 (55)
	Male	59 (38)	12 (28.6)	16 (38)	10 (41.7)	12 (44.4)	9 (45)
Median Age		60	53.5	62.5	52.5	61	67
[IQR]		[47, 69]	[42, 65]	[55, 71]	[39, 61]	[52, 71]	[60, 73]
Stage	I	34 (21.9)	11 (26.2)	9 (21.4)	0	6 (22.2)	8 (40)
(AJCC 8th Edition)	II	44 (28.4)	15 (35.7)	14 (33.3)	4 (16.7)	8 (29.6)	3 (15)
	III	21 (13.5)	4 (9.5)	12 (28.6)	3 (12.5)	0	2 (10)
	IV	54 (34.8)	12 (28.6)	5 (11.9)	17 (70.8)	13 (48.1)	7 (35)
	Unknown	2 (1.3)	0	2 (4.8)	0	0	0
Perineural Growth	Yes	100 (64.5)	29 (69)	32 (76.2)	16 (66.7)	16 (59.3)	7 (35)
	No	52 (33.5)	12 (28.6)	10 (23.8)	8 (33.3)	10 (37)	12 (60)
	Unknown	3 (1.9)	1 (2.4)	0	0	1 (3.7)	1 (5)
Smoking	Ever	81 (52.3)	20 (47.6)	24 (57.1)	15 (62.5)	15 (55.6)	7 (35)
	Never	72 (46.5)	22 (52.4)	16 (38.1)	9 (37.5)	12 (44.4)	13 (65)
	Unknown	2 (1.3)	0	2 (4.8)	0	0	0

**Table 2:** Patients treated with curative intent and tumour characteristics.

		Total (%)	Parotid Gland (%)	Submandibular Gland (%)	Nasal Cavity & Paranasal Sinuses (%)	Oral Cavity (%)	Other Sites (%)
Number of patients		142	41	38	21	25	17
Gender	Female	88 (62)	30 (73.2)	23 (60.5)	12 (57.1)	13 (52)	10 (58.8)
	Male	54 (38)	11 (26.8)	15 (39.5)	9 (42.9)	12 (48)	7 (41.2)
Median Age [IQR]		58.5 [46, 68]	53 [42, 64]	59.5 [49, 68]	53 [40, 60]	61 [52, 70]	66 [58, 72]
Age Group	<58.5	71 (50)	25 (61)	18 (47.4)	14 (66.7)	9 (36)	5 (29.4)
	≥58.5	71 (50)	16 (39)	20 (52.6)	7 (33.3)	16 (64)	12 (70.6)
Stage	I	34 (23.9)	11 (26.8)	9 (23.7)	0 (0)	6 (24)	8 (47.1)
	II	43 (30.3)	15 (36.6)	14 (36.8)	4 (19)	7 (28)	3 (17.6)
	III	20 (14.1)	4 (9.8)	11 (28.9)	3 (14.3)	0 (0)	2 (11.8)
	IV	45 (31.7)	11 (26.8)	4 (10.5)	14 (66.7)	12 (48)	4 (23.5)
Perineural Growth	Yes	95 (66.9)	28 (68.3)	31 (81.6)	13 (61.9)	16 (64)	7 (41.2)
	No	46 (32.4)	12 (29.3)	7 (18.4)	8 (38.1)	9 (36)	10 (58.8)
	Unknown	1 (0.7)	1 (2.4)	0 (0)	0 (0)	0 (0)	0 (0)
Smoking	ever	75 (52.8)	19 (46.3)	22 (57.9)	13 (61.9)	14 (56)	7 (41.2)
	never	67 (47.2)	22 (53.7)	16 (42.1)	8 (38.1)	11 (44)	10 (58.8)
Metastasis at Diagnosis	no	140 (98.6)	40 (97.6)	38 (100)	21 (100)	25 (100)	16 (94.1)
	yes	2 (1.4)	1 (2.4)	0 (0)	0 (0)	0 (0)	1 (5.9)
Surgery	no	14 (9.9)	5 (12.2)	0 (0)	2 (9.5)	3 (12.0)	4 (23.5)
	yes	128 (90.1)	36 (87.8)	38 (100)	19 (90.5)	22 (88)	13 (76.5)
Radical Surgery	no	97 (75.8)	30 (83.3)	28 (73.7)	15 (78.9)	19 (86.4)	5 (38.5)
	yes	31 (24.2)	6 (16.7)	10 (26.3)	4 (21.1)	3 (13.6)	8 (61.5)
RT	no	10 (7)	2 (4.9)	3 (7.9)	1 (4.8)	0 (0)	4 (23.5)
	yes	132 (93.0)	39 (95.1)	35 (92.1)	20 (95.2)	25 (100)	13 (76.5)
CRT	no	105 (73.9)	34 (82.9)	30 (78.9)	11 (52.4)	18 (72)	12 (70.6)
	yes	37 (26.1)	7 (17.1)	8 (21.1)	10 (47.6)	7 (28)	5 (29.4)
Induction ChT	no	137 (96.5)	40 (97.6)	37 (97.4)	20 (95.2)	24 (96)	16 (94.1)
	yes	5 (3.5)	1 (2.4)	1 (2.6)	1 (4.8)	1 (4)	1 (5.9)
Surgery + RT/CRT	No	24 (16.9)	7 (17.1)	3 (7.9)	3 (14.3)	3 (12.0)	8 (47.1)
	Yes	118 (83.1)	34 (82.9)	35 (92.1)	18 (85.7)	22 (88.0)	9 (52.9)
Surgery + CRT		29 (24.6)	5 (14.7)	8 (22.9)	8 (44.4)	5 (22.7)	3 (33.3)
Surgery + RT		89 (75.4)	29 (85.3)	27 (77.1)	10 (55.6)	17 (77.3)	6 (66.7)

## Data Availability

The data presented in this study are available on request from the corresponding author, but cannot be made publicly available due to Swedish laws on personal confidential information.

## Supplementary Materials

The following supporting information can be downloaded at: https://www.mdpi.com/article/10.3390/cancers15051499/s1, Supplementary Table S1. 1-, 5-, 10-, and 15-year disease free survival (DFS) and overall survival (OS). Supplementary Table S2. Univariable analysis of disease-free survival (DFS) and overall survival (OS) in patients treated with curative intent. Supplementary Figure S1. Overall survival (OS) of patients treated with curative intent separated for treatment modality, multimodal treatment (Yes) and surgery or RT w/o ChT (No). Supplementary Figure S2. Overall survival (OS) of patients treated with curative intent separated for treatment modality, surgery + PORT and surgery + postoperative CRT. Supplementary Figure S3. Overall survival of patients treated with curative intent independent of treatment modality, depending on gender (A), age (B) and smoking (C). Supplementary Figure S4. Overall survival of patients with tumours staged I–IV (A) or with tumours staged I and II vs. III and IV (B). Supplementary Figure S5. Multivariable analysis of overall survival (OS) in patients treated with curative intent.
