# Supplementary material for: Adenoid Cystic Carcinoma (AdCC): A Clinical Survey of a Large Patient Cohort
Source: Cancers (Basel). 2023 Feb 27;15(5):1499. doi: 10.3390/cancers15051499 (PMC10000643; doi:10.3390/cancers15051499)
Supplement: Supplementary file 1 [file cancers-15-01499-s001.zip › cancers-2228322-supplementary.pdf]

Supplementary Table S1. 1-, 5-, 10-, and 15-year disease free survival (DFS) and overall survival (OS)

		**1y**	**95% CI**		**5y**	**95% CI**		**10y**	**95% CI**		**15y**	**95% CI**	
DFS	All	95.7	92.5	99.1	64.9	57.1	73.9	49.6	41.1	59.9	37.7	28.5	49.9
OS	All	98.6	96.6	100.0	83.5	77.3	90.3	59.4	50.4	70.0	42.5	32.4	55.8
DFS	Mult trt no	91.7	81.3	100.0	37.0	20.9	65.6	14.8	4.4	50.0			
DFS	Mult trt yes	96.6	93.3	99.9	70.7	62.5	80.1	56.4	47.1	67.4			
OS	Mult trt no	95.8	88.2	100.0	60.9	43.8	84.8	36.5	18.5	72.1			
OS	Mult trt yes	99.1	97.5	100.0	88.5	82.6	94.9	64.2	54.6	75.5			
DFS	Female	96.6	92.9	100.0	65.7	56.0	77.0	50.6	40.3	63.5	38.6	27.7	53.8
DFS	Male	94.3	88.3	100.0	63.7	51.1	79.4	47.3	33.5	66.8	35.0	20.3	60.6
DFS	<58.5 age	95.7	91.1	100.0	67.3	56.6	80.0	54.5	42.7	69.4	43.7	30.7	62.2
DFS	58.5+ age	95.7	91.1	100.0	62.8	51.9	76.0	45.4	34.0	60.5	32.7	21.2	50.4
DFS	Ever	94.6	89.5	99.9	66.4	56.0	78.7	47.6	36.4	62.3	32.7	21.8	49.2
DFS	Never	97.0	93.0	100.0	63.3	52.0	77.1	52.2	40.2	67.7	47.4	34.4	65.3
OS	Female	98.9	96.7	100.0	86.6	79.4	94.3	60.2	49.3	73.5	46.0	34.0	62.0
OS	Male	98.1	94.5	100.0	78.3	67.1	91.2	58.6	44.2	77.6	36.2	20.4	64.2
OS	<58.5 age	98.6	95.8	100.0	91.1	84.5	98.2	69.7	58.0	83.7	52.7	38.4	72.3
OS	58.5+ age	98.6	95.9	100.0	75.7	65.7	87.3	49.0	36.7	65.5	32.6	20.5	52.1
OS	Ever	97.3	93.7	100.0	85.1	76.9	94.1	57.9	46.2	72.7	40.9	28.9	58.0
OS	Never	100	100.0	100.0	81.7	72.5	92.2	61.7	48.8	77.8	44.4	28.2	70.0
DFS	Perineural yes	96.8	93.3	100.0	63.8	54.0	75.6	50.4	39.8	63.8	37.3	26.0	53.3
DFS	Perineural no	93.5	86.6	100.0	65.0	52.5	80.4	46.7	33.7	64.7	41.5	27.8	61.9
OS	Perineural yes	99.0	96.9	100.0	89.1	82.5	96.2	57.8	46.2	72.3	42.7	30.5	59.7
OS	Perineural no	97.8	93.7	100.0	73.4	61.6	87.6	59.3	45.8	76.8	39.7	24.4	64.7
DFS	I	100.0	100.0	100.0	88.1	77.9	99.8	76.6	62.7	93.7			
DFS	II	95.4	89.3	100.0	80.1	68.6	93.5	63.5	49.1	82.1			
DFS	III	89.5	76.7	100.0	42.2	23.3	76.5	28.1	12.4	63.9			
DFS	IV	95.5	89.6	100.0	41.1	28.3	59.7	21.8	11.0	43.2			
OS	I	100.0	100.0	100.0	96.7	90.5	100	79.7	65.1	97.5			
OS	II	97.7	93.3	100.0	84.4	74.2	96.6	66.6	51.8	85.7			
OS	III	100.0	100.0	100.0	81.2	64.0	100.0	43.1	23.2	80.1			
OS	IV	97.7	93.4	100.0	72.5	69.8	87.8	42.6	28.0	64.9			
DFS	I-II	97.4	93.9	100.0	83.6	75.5	92.6	69.2	58.6	81.8	56.0	43.5	72.0
DFS	III-IV	93.7	87.9	99.9	41.3	30.0	56.9	23.9	14.1	40.3	9.5	2.0	44.7
OS	I-II	98.7	96.2	100.0	90.0	83.3	97.3	72.3	61.3	85.2	61.3	48.6	77.3
OS	III-IV	98.4	95.4	100.0	75.0	64.4	87.3	42.7	30.1	60.5	16.9	6.8	42.0
DFS	Gl. parotidea	100.0	100	100.0	81.1	69.3	94.9	74.0	60.5	90.6			
DFS	Gl. submandibularis	91.0	83.6	100.0	68.3	54.4	85.9	50.6	35.6	71.8			

DFS	N. cavity & para. sinuses	90.5	78.8	100.0	42.0	24.3	72.6	7	1.1	45.5			
DFS	Oral Cavity	96.0	88.6	100.0	61.2	44.2	84.8	50.5	33.1	77.0			
OS	Gl. parotidea	100.0	100.0	100.0	94.3	87.0	100.0	78.7	64.7	95.7	78.7	64.7	95.7
OS	Gl. submandibularis	97.4	92.4	100.0	82.4	70.5	96.3	47.7	31.9	71.3	39.8	23.2	68.1
OS	N. cavity & para. sinuses	95.2	86.6	100.0	73.7	56.2	96.7	53.6	34.1	84.3	33.5	16.6	67.6
OS	Oral Cavity	100.0	100.0	100.0	82.5	68.3	99.7	57.8	38.7	86.3	19.6	6.4	60.3
DFS	Other sites	95.1	89.8	100.0	53.7	42.0	68.8	33.8	22.5	50.9	17.9	8.3	38.7
DFS	Salivary glands	96.2	92.1	100.0	73.5	63.9	84.7	61.3	50.4	74.5	51.9	39.5	68.1
OS	Other sites	98.4	95.2	100.0	76.8	66.4	88.8	54.2	41.3	71.1	23.6	12.2	45.6
OS	Salivary glands	98.8	96.4	100.0	88.7	81.5	96.4	63.6	52.0	77.8	59.9	47.4	75.7
DFS	Surgery + CRT	93.1	84.3	100.0	62.1	46.7	82.5	44.8	29.9	67.1			
DFS	Surgery + RT	97.7	94.6	100.0	73.7	64.4	84.5	61.5	50.7	74.5			
OS	Surgery + CRT	96.6	90.1	100.0	79.3	65.9	95.5	52.9	36.3	77.3			
OS	Surgery + RT	100.0	100.0	100.0	92.0	86.0	98.4	68.6	57.6	81.7			

Supplementary Table S2. Univariable analysis of disease-free survival (DFS) and overall survival (OS) in patients treated with curative intent

	Disease-free survival (DFS)				Overall survival (OS)			
	**HR**	**95% CI**		**p-value**	**HR**	**95% CI**		**p-value**
**Sex**								
Female	1.00	reference			1.00	reference		
Male	1.16	0.71	1.91	0.55	1.28	0.74	2.22	0.374
**Age groups**								
<58.5	1.00	reference			1.00	reference		
58.5+	1.28	0.79	2.08	0.311	1.87	1.08	3.24	0.025
**Smoking**								
Ever	1.00	reference			1.00	reference		
Never	0.82	0.50	1.34	0.436	0.91	0.53	1.57	0.737
**Stage**								
I-II	1.00	reference			1.00	reference		
III-IV	3.89	2.32	6.53	<0.001	2.94	1.69	5.12	<0.001
**Perineural invastion**								
No	1.00	reference			1.00	reference		
Yes	0.97	0.59	1.60	0.914	0.93	0.54	1.61	0.802
**Multimodal treatment**								
No	1.00	reference			1.00	reference		
Yes	0.31	0.18	0.55	<0.001	0.31	0.16	0.58	<0.001
**Sides**								
Salivary glands	1.00	reference			1.00	reference		
Other subsides	2.51	1.53	4.10	<0.001	2.06	1.20	3.56	0,009

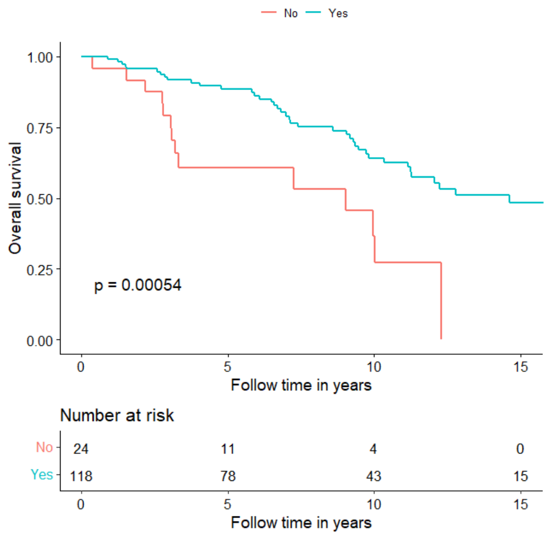

Supplementary Figure S1. Overall survival (OS) of patients treated with curative intent separated for treatment modality, multimodal treatment (**Yes**) and surgery or RT w/o ChT (**No**).

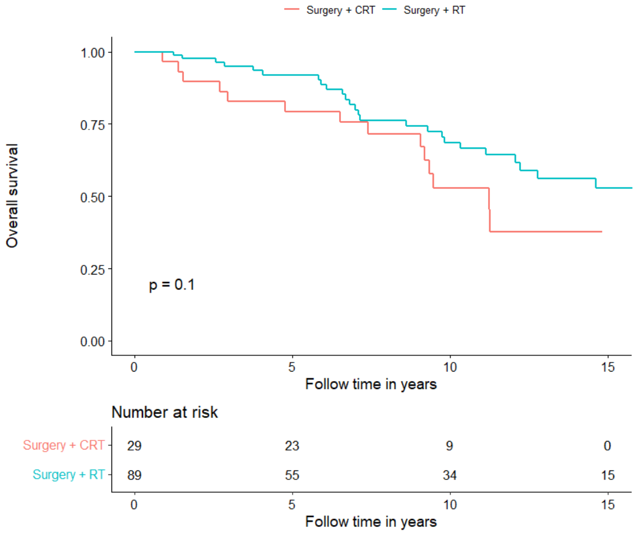

Supplementary Figure S2. Overall survival (OS) of patients treated with curative intent separated for treatment modality, surgery + PORT and surgery + postoperative CRT.

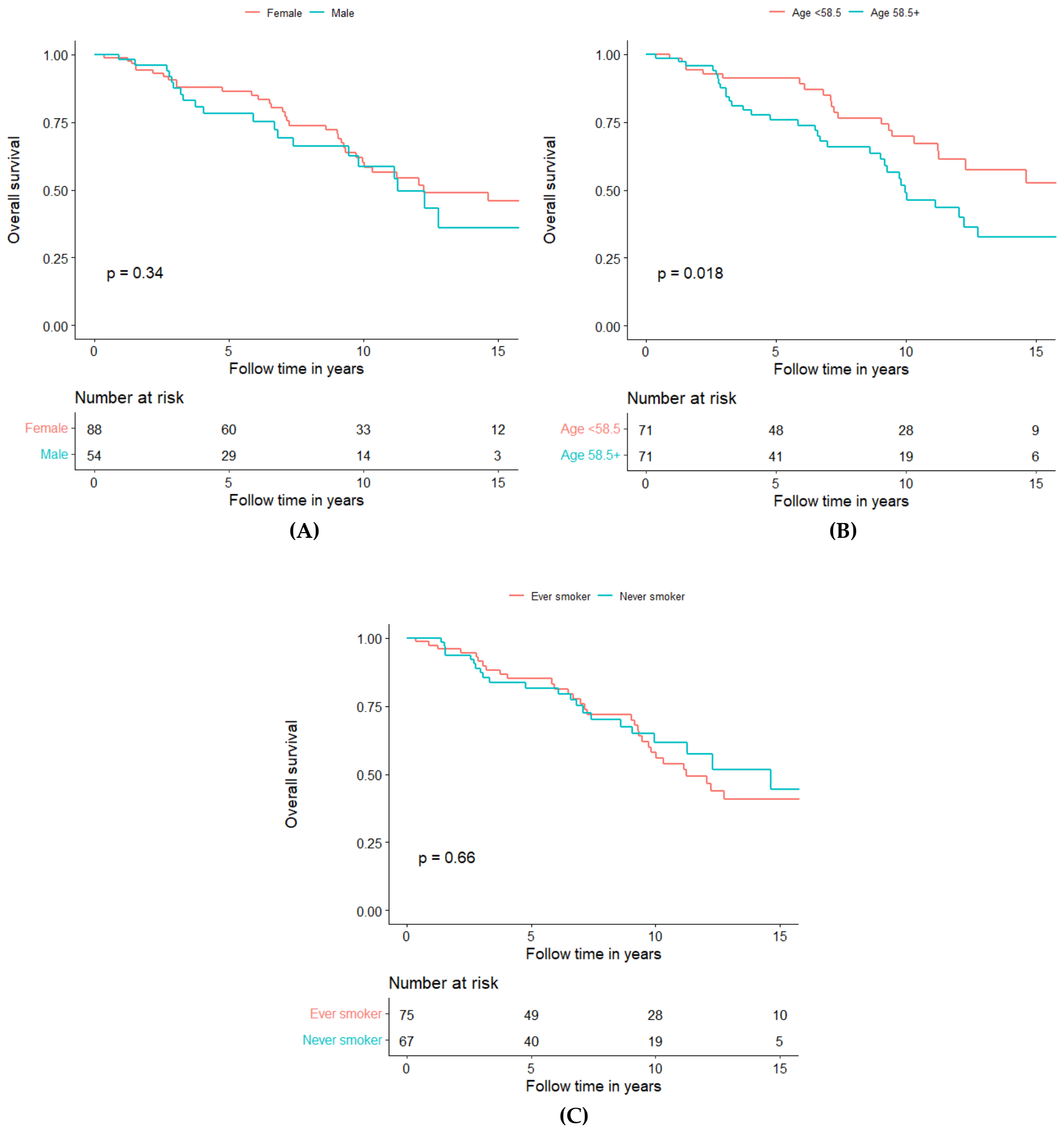

Supplementary Figure S3. Overall survival of patients treated with curative intent independent of treatment modality, depending on gender (**A**), age (**B**) and smoking (**C**).

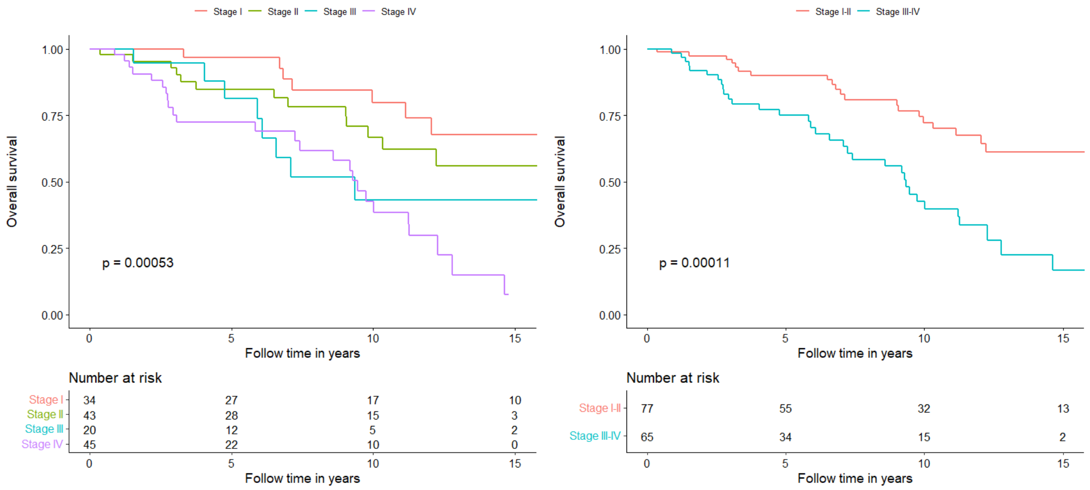

(A) **(B)**

Supplementary Figure S4. Overall survival of patients with tumours staged I-IV (**A**) or with tumours staged I and II vs. III and IV (**B**).

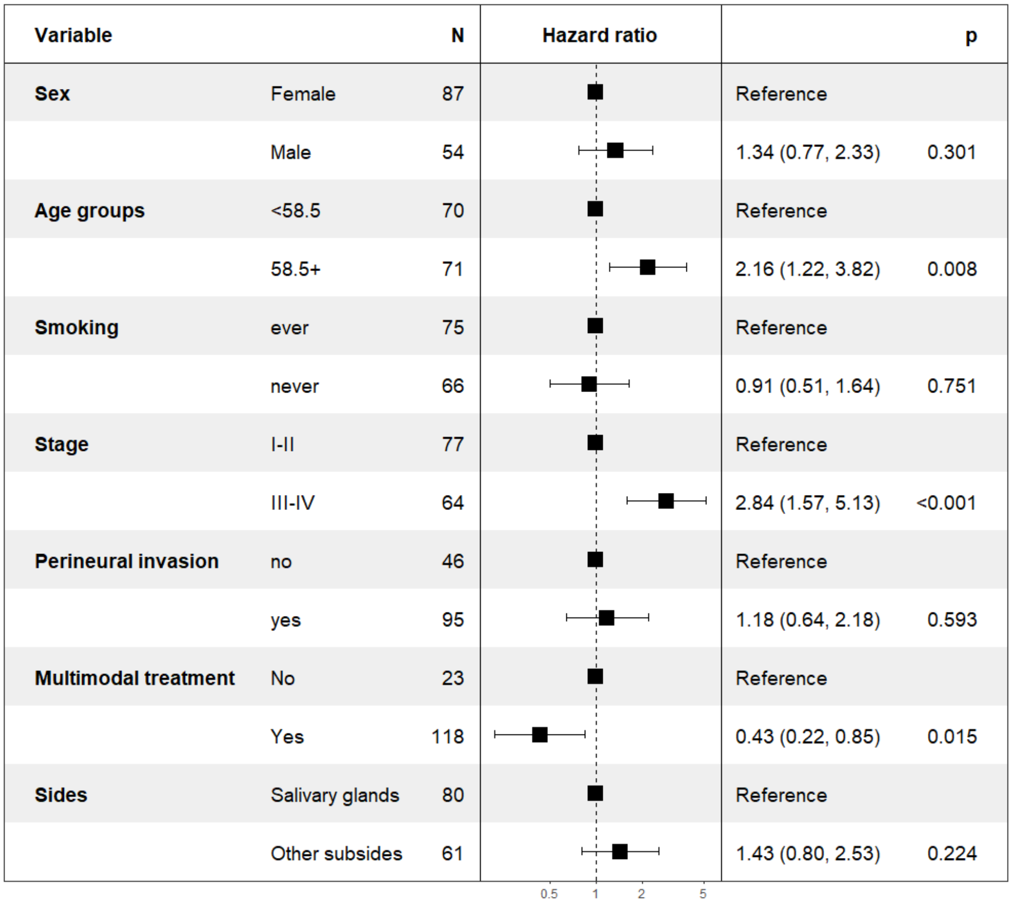

Supplementary Figure S5. Multivariable analysis of overall survival (OS) in patients treated with curative intent.
